# 2D Spatially-Resolved Depth-Section Microfluidic Flow Velocimetry Using Dual Beam OCT

**DOI:** 10.3390/mi11040351

**Published:** 2020-03-27

**Authors:** Jonathan M. Hallam, Evangelos Rigas, Thomas O. H. Charrett, Ralph P. Tatam

**Affiliations:** Centre for Engineering Photonics, Cranfield University, Cranfield MK43 0AL, Bedfordshire, UK; Jonathan.Hallam@cranfield.ac.uk (J.M.H.); e.rigas@cranfield.ac.uk (E.R.); t.charrett@cranfield.ac.uk (T.O.H.C.)

**Keywords:** optical coherence tomography (OCT), interferometry, microfluidics, flow measurement, particle image velocimetry (PIV)

## Abstract

A dual beam optical coherence tomography (OCT) instrument has been developed for flow measurement that offers advantages over microscope derived imaging techniques. It requires only a single optical access port, allows simultaneous imaging of the microfluidic channel, does not require fluorescent seed particles, and can provide a millimetre-deep depth-section velocity profile (as opposed to horizontal-section). The dual beam instrument performs rapid re-sampling of particle positions, allowing measurement of faster flows. In this paper, we develop the methods and processes necessary to make 2D quantitative measurements of the flow-velocity using dual beam OCT and present exemplar results in a microfluidic chip. A 2D reference measurement of the Poiseuille flow in a microfluidic channel is presented over a spanwise depth range of 700 μm and streamwise length of 1600 μm with a spatial resolution of 10 μm, at velocities up to 50 mm/s. A measurement of a more complex flow field is also demonstrated in a sloped microfluidic section.

## 1. Introduction

As the microfluidic field has matured in academia and industry, there is an increasing need for robust and straightforward imaging tools [1] for live monitoring during industrial or scientific bio-processes [2], to observe experiments in a research environment [3], and for characterising flows during the prototyping and development of new microfluidic designs [1]. Most microfluidic systems involve flows, for processes as diverse as mixing, separation, dropletisation, and controlled dispensing and cell delivery [2,3,4,5,6]. Current microfluidic applications are primarily in the biochemical and biomedical areas, including the growth and support of artificial organs, “lab-on-a-chip” bio-analysis for point-of-care diagnostics, chemical and food processing, encapsulation for drug manufacture and delivery, drug discovery and medical research, as well as environmental monitoring and on-board cooling for 3D microprocessors [3,4,5,6,7,8].

In these applications, the measurement of flow velocities from micrometres-per-second to metres-per-second is potentially of interest [2], with lower velocities of interest typically being associated with the requirement for the spatial resolution to be in at least two dimensions. Existing measurement techniques have limitations, such as the requirement for multiple optical access ports in stereoscopic or volumetric methods [9] or the use of fluorescent seed particles typically required by microscope-derived techniques [8,10], which also tend to provide only horizontal section measurement. Doppler OCT has measured 30 mm/s flow velocities, but is only capable of doing so for velocity components along the beam axis [11]. Confocal microscopy can acquire many horizontal sections to capture a volume, but the time required to do so implies maximum flow velocities of millimetres-per-second [1]. Optical coherence tomography (OCT) has been used to measure flow at velocities of a few millimetres-per-second [12,13] using a single beam creating a depth-priority image. En face OCT, creating a lateral-priority image (which with dynamic focusing can be in-focus at all depths, sharing some similarities with confocal microscopy) requires 0.5 s to create an image. This is too slow for most flow situations, but it may be possible to track particles in the lateral-plane only without scanning all depths using this technique in which case scan rates of 500 Hz can be achieved [14]. A commercial multi-beam OCT system exists [15,16] for en face OCT with each beam being in-focus at a different depth, which would in principle allow for faster image capture [17].

We previously reported a novel dual beam optical coherence tomography (OCT) instrument [18] that overcomes some of these limitations: the instrument requires only a single optical access port (in this case, above the microfluidic chip), allows simultaneous imaging of the microfluidic channel, does not require fluorescent seed particles, and can provide a depth-section velocity profile (as opposed to horizontal-section) at full millimetre depth through a microfluidic chip. This instrument uses two beams to re-image the same plane rapidly (analogous to double-exposure camera-based PIV) and hence measure high flow velocities [19], whereas the Vivosight system [15,16] uses multiple beams, each focused at a different depth to improve the lateral resolution and depth of field. Our instrument has been used to make 1D velocity profiles of the flow spanwise across a straight channel with velocities of up to 1 m/s. At the associated 4th Microfluidic Handling Systems conference [20], preliminary work was presented showing the visualisation of two-dimensional microfluidic flows using this instrument.

In this paper, we develop the methods and processes necessary to make 2D quantitative measurements of the flow-velocity using dual beam OCT and present exemplar results in a microfluidic chip. A 2D reference measurement of the Poiseuille flow in a trapezoidal microfluidic channel is presented over a spanwise depth range of 700 μm and streamwise length of 1600 μm with a spatial resolution of 10 μm, at velocities up to 50 mm/s. The measurement of a more complex flow field is also demonstrated in a sloped microfluidic section.

## 2. Materials and Methods

### 2.1. Dual Beam Optical Coherence Tomography Instrument

**Figure 1:**
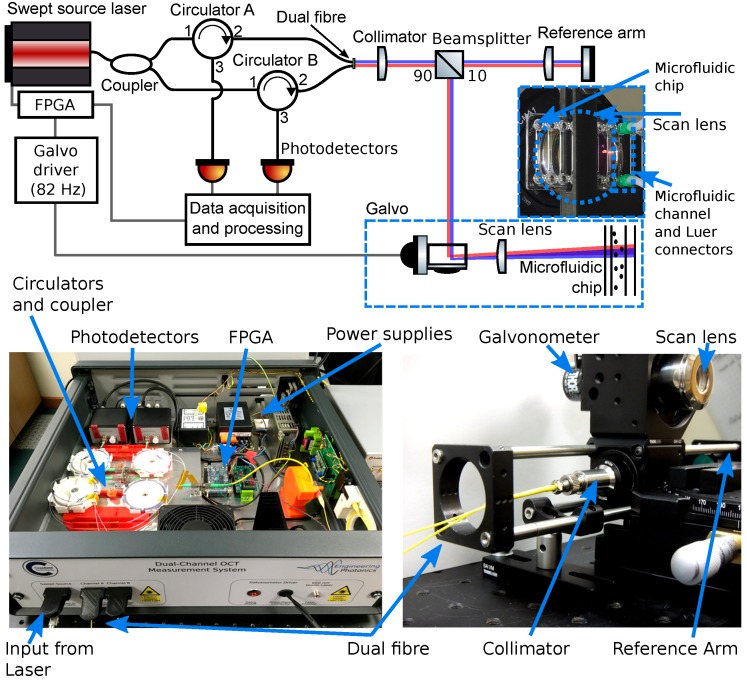
(**Top**) Schematic of the dual beam optical coherence tomography (OCT) instrument. Red and blue indicate the light from each of the single mode fibres contained within the dual optical fibre end propagating in free space (both beams are at the same wavelength). The beamsplitter uses 90% of the input light for the microfluidic chip and 10% for the shared reference arm of the interferometers. The microfluidic chip (containing 16 copies of the utilised microfluidic channel) through which the scan lens can be seen is pictured on the right. (**Bottom left**) Labelled picture of the custom-assembled instrument in a portable enclosure. (**Bottom right**) Labelled picture of the scan head without the microfluidic chip. Reproduced with permission from [18].

The dual beam OCT instrument is shown schematically in Figure 1 and was described in more detail in [18]. The instrument was custom assembled from commercially available optical components, excluding the dual optical fibre end, which was manufactured in our laboratories. In this system, two parallel OCT interferometers were created using a single laser and reference arm, with the measurement arm illuminating the microfluidic chip with a small spatial separation of 180 μm.

**Figure 2:**
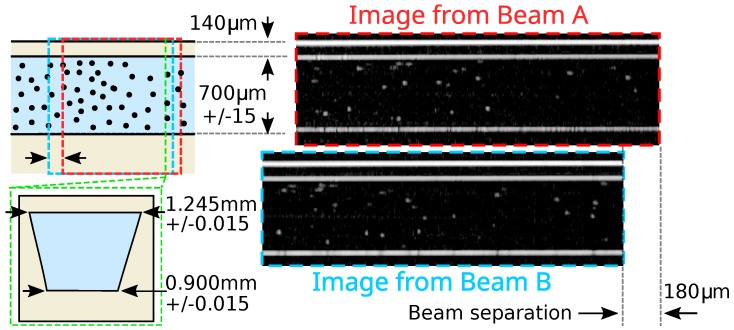
Schematic of the straight channel section of the microfluidic chip with the area imaged by Beams A and B of the dual beam OCT instrument marked and images from the dual beam OCT instrument with the appropriate beam separation. A cross-section of the microfluidic channel is shown in the lower left.

**Figure 3:**
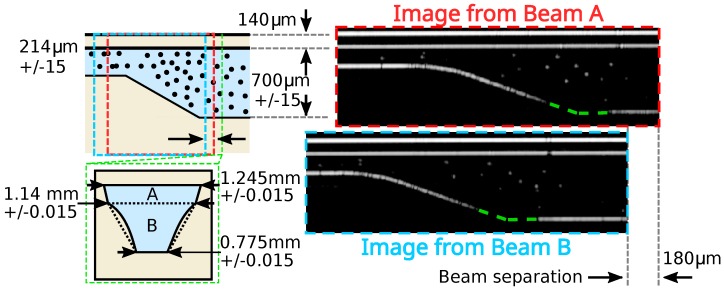
Schematic of the sloped section of the microfluidic chip with the area imaged by Beams A and B of the dual beam OCT instrument marked with blue and red outlines. The actual OCT images are shown with matching outline colours. The superimposed green dashed line marks the location of the slope. The spanwise width cross-section of the microfluidic channel is shown in the lower left outlined in green, including the PMMA slumping around the base of the channel.

The dual beams were created by coupling light from a solid-state swept-source laser (1526–1608 nm, 9 mW output power) into separate, but closely adjacent optical fibres within a custom-made dual optical fibre end as described in [18]. This dual optical fibre end was integrated into an interferometric sensing head that addressed the microfluidic chip (at present, through its top surface). This configuration allowed the simultaneous measurement of two spatially offset depth profiles into the microfluidic chip. A beamsplitter within the sensing head used 90% of the input light for the microfluidic chip arm of the interferometer in order to balance the returning optical power, since the microfluidic chip reflected relatively little light compared to the reference arm. A galvanometer was then used to scan the beam pair through a focusing scan lens and across the chip, allowing two-dimensional depth-section images of flow in the vertical plane to be measured. The focusing scan lens created a beam waist of 20 μm in diameter (corresponding to lateral imaging resolution), resulting in a Rayleigh range of 0.8 mm (within which the beam size remained within a factor of $\sqrt{2}$ of its original size). The axial imaging resolution of 15 μm in air (or 10 μm in the fluid) was principally determined by the swept wavelength of the laser (1526–1608 nm) [21]. These resolutions applied to the OCT images shown in Figure 2 and Figure 3 and not to the spatial resolution of the calculated velocity vectors determined by the particle tracking velocimetry analysis. Except in specific cases when determining the refractive index and depth to 4 significant figures [22], dispersion effects can typically be ignored in OCT imaging.

The optics necessary for separation (and later detection) of the light were custom assembled into a portable enclosure that also served to isolate the instrument from changes in the environment. Optical circulators redirected the reflected light to two photo-detectors from which the data were acquired by a high-rate data-acquisition card (12 bit resolution, 500 MS/s) with the optical coherence tomography images reconstructed from the photo-detector signals using the control/capture PC, as detailed in [18]. This data acquisition rate was sufficiently high that each pixel in the OCT images was approximately 3 μm in length.

This instrument was used to measure fluid flow through straight and sloped microfluidic channel formations using only a single optical access point through the “upper” surface of the microfluidic chip. A standard reaction chamber chip (Microfluidic Chipshop Jena GmBH, Fluidic 558, Jena, Germany) contained both formations and was used as the test object. This chip was manufactured from PMMA. The sloped section rose from a (specified) full depth of 700 μm (measured to be accurate to ±15 μm) to 214 μm. The spanwise width narrowed from a (specified) width of 1.25 mm (measured to be 1.245 mm) at the top of the channel to 0.90 mm at the bottom (with additional narrowing to 0.77 mm around the base of the slope, possibly due to PMMA flow during the injection moulding process) [23]. Measurements were accurate to 15 μm.

Flexible tubing of internal diameter 0.78 mm was attached to a 10 mL plastic syringe driven by a Nexus 3000 (Chemyx, Stafford, TX, USA) syringe pump, with the other microfluidic chip port connected to a reservoir. A colloidal suspension of 10 μm diameter latex particles was prepared at a density of 1.05 g/mL (matched to the particles so they would remain in suspension for a prolonged period, although in practice, the particles were slightly buoyant). This particle size was chosen for the reasonable visibility of individual particles as compared to the 20 μm beam waist (each particle filled approximately one-fourth of the resolved volume) and sufficient oversampling of particles as compared to the 3 μm pixel size in the OCT images, ensuring that the particles did not appear as single pixel dots that could be confused with system noise. The 3 μm particles of the same type were also tested and could not be clearly imaged. It was unlikely that particles small enough to exhibit significant Brownian motion in the approximately 1 ms between Beam A and Beam B could be individually imaged using OCT.

### 2.2. OCT Imaging of Straight and Sloped Channel Sections of the Microfluidic Chip

OCT images of the straight channel section of the microfluidic chip produced by interferometry are shown in Figure 2. The interferometry occurred between the portion of reflected (or scattered) light that returned from the microfluidic chip and the light from the reference arm of the interferometer. A pair of images was produced, one for each of the dual beams, with the separation of the images determined by the spacing of the fibres in the dual optical fibre end.

The flat upper surface of the microfluidic chip, the flat upper surface of the microfluidic channel, and the flat portions of the lower surface of the microfluidic channel could clearly be seen, as they reflected light directly back to the dual optical fibre end. The spherical particles in suspension within the microfluidic channel scattered light in all directions, a sufficient portion of which returned to the dual optical fibre end to allow the particles to be imaged, and these could be seen as the bright specks within the channel.

Some particles appeared less intensely than others, likely because they lay only partially within the plane of the beam scan. Particles moving quickly across the image only interacted briefly with the beams performing the OCT scan and therefore appeared compressed. This effect could be seen in middle of the microfluidic channel (the flow at the centre of a channel was faster than near the walls) [24]. Slightly fewer particles appeared at the bottom of the channel than at the top. This was due in part to the distance from the beam focus and partly due to absorption of the laser light by the fluid. Significant effort has been made in recent years to restore at-focus resolution across the full depth of OCT images mainly de-convolving the point-spread-function of the OCT system with the image [25]. In principle, such techniques might also be used to restore the intensity of particles partially out of the beam-scan plane. Neither technique was applied here; instead, a particle identification approach (using widths appropriate for the point-spread-function) was used, as detailed in Section 2.3.

OCT images of the sloped section are shown in Figure 3. In this case, the lower surface of the microfluidic channel incorporated a smooth sloped section that was not clearly seen because it reflected light away from the optical fibre end, and this is shown in Figure 3 with a green dashed line.

### 2.3. Particle Flow Tracking

The particles were imaged and tracked in order to determine the flow velocity. The particle identification and flow tracking were performed using the open source trackpy 0.3.3 library [26]. The area of interest was windowed to within the microfluidic channel. Insufficiently intense particles were rejected using the following method. First, the OCT images were re-scaled in brightness to maximise contrast following the method of [27]. Then, the trackpy software searched for particles within the image, allowing a size of 5 pixels vertically by 3 pixels horizontally with a minimum separation between particles of 3 pixels. Particles had to exceed a minimum mass value of 0.4 related to the sum of their brightness in all pixels in which they appeared. Additionally, particles had to appear brighter than 74% of the image as a whole. These values were determined by trial-and-improvement testing on a sampling of images from the dataset, and the implementation can be found in the supplementary materials.

The process of particle identification was repeated for 300 captured pairs of images corresponding to a 3.65 s measurement, which was a limitation of the current data acquisition system. It was not possible to use particle intensity to match particles between the images as particle brightness could change between the images, either due to misalignment between the beams or the particles shifting slightly with respect to the plane of the measurement between the beams. Therefore, the software took the identified particle positions alone for the two images and searched for a matrix of velocity vectors that connected those positions. The flow was assumed constant over the 3.65 s duration of the measurement, so similar velocity matrices were expected to match for all pairs of images, and this condition was imposed by the software.

A predicted velocity vector matrix anticipating parabolic Poiseuille flow streamwise [24] along the microfluidic channel was used as a starting point for the velocity vector search (horizontally in Figure 2 and Figure 3) to account for the straight sections of the channel. However, the software was permitted to diverge strongly from this starting velocity vector since it was expected that the sloped section of the channel would induce flow velocity vectors with vertical components. Although it would have been possible to provide a bespoke flow predictor for the starting estimate in the flow tracking, this was not desirable as this required a degree of foreknowledge not likely to be present and an impractical degree of data analysis effort, for many realistic scenarios.

### 2.4. Spatial Resolution, Velocity Error, and Field-of-View

**Figure 4:**
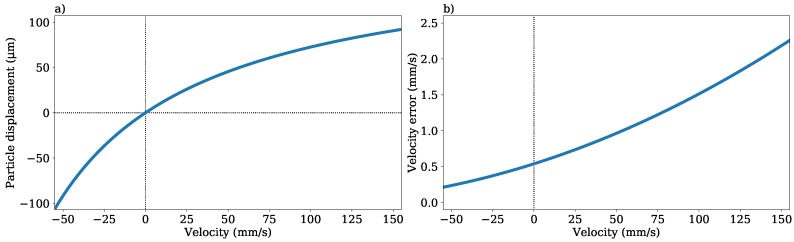
(**a**) Particle displacement between the image frames plotted against the particle velocity. (**b**) Velocity error per bin plotted against the particle velocity, assuming a half-pixel particle position error and 100 particles in each bin.

The length of the determined particle displacement vector provided a measure of the spatial resolution achievable and was dependant on the flow velocity, as shown in Figure 4a. For example, at a particle velocity equal to the beam-scan velocity of 148 mm/s, the particle displacement was 90 μm, half the beam separation (because the moving particle had met the oncoming second beam halfway). Likewise, since the scanning dual beams and the particles were both moving and the direction of the beam scan should be chosen so as to be opposite the main direction of flow [18], the resolution with which the magnitude of a velocity vector could be determined (the velocity error) was itself dependant on the flow velocity. Assuming an uncorrelated half-pixel error in the position determination of a particle and 100 particles per bin (with the error falling as $\sqrt{N}$ where *N* is the number of particles [28]), the velocity error could be plotted against the velocity in Figure 4b. It could be seen that the error would increase non-linearly with particle velocity in the direction opposite the beam. When the particle velocity became negative, travelling in the same direction as the beam scan, the particle displacement approached infinity (a particle travelling at the same velocity as the beams would never be imaged by them) and the velocity error approached zero (a half-pixel position error divided by an infinitely long particle displacement).

Unlike traditional single beam OCT systems, there was no explicit link between the size of the image and the measurable velocities [12,13], because the second beam imaged the particle without having to wait for the next scan. The overlap between the images from the two beams had to be substantial, in this case a 1.8 mm scan with a beam separation of 180 μm. Given the fixed half-pixel error in particle position determination, it may be desirable to increase the scan resolution to reduce the size (in micrometres) represented by a pixel in the OCT images. This could be done either by reducing the length of the scan or increasing the number of laser wavelength sweeps in a single image (which given the fixed laser scan rate of 96 kHz implied reducing the frame rate). The system could be configured in either way, and the appropriate settings for the measurement should be chosen. In this case, a pixel width of ≈3 μm was used to minimise the error, given the 20 μm diameter of the beam waist and the 10 μm size of the particles [28].

## 3. Results

### 3.1. Flow Velocimetry for the Straight Channel Section of the Microfluidic Chip

To provide a reference measurement, spatially-resolved flow velocimetry was performed on the well-defined case of a straight channel, where parabolic Poiseuille flow was expected [24,29]. The time-resolved velocity vectors previously determined for a single 3.65 s acquisition were merged to form a set of 16 acquisition with a total time of 58.4 s captured over approximately a 10 min duration (it was not currently possible to capture a continuous acquisition due to buffering in the data acquisition card). The syringe pump was programmed to provide constant flow over this period.

The velocity vectors (for all times of the combined acquisitions) were binned in the streamwise direction according to their starting position, with a bin length of 10 μm, setting the spatial resolution of the calculated velocity vectors (long velocity vectors would cross multiple bins, but to prevent over-weighting, these vectors were assigned to the bin in which they originated). Particle tracking velocimetry techniques allowed for a spatial resolution of velocities as small as the particle size (10 μm) [30], provided the Stokes number was appropriate (so the particles followed the flow), which depended on the conditions of the flow [28]. This implied that high speed features such as vortexes would not be detected, even if these features were larger than the spatial resolution of the calculated velocity vectors. Typical microscope-based micro-PIV systems offered resolutions of around 5 μm, although usually dealing with sub-millimetre-per-second velocities [28] (rather than centimetre-per-second as here) and much smaller particles. Bins were cut from the streamwise end of the channel corresponding to the region where the dual beams did not overlap (with a slight extension to account for the fact that a particle must be detected at both the start and end of a velocity vector). Fewer vectors were present at the bottom of the channel due to reduced particle detection caused by defocus away from the beam waist and absorption of the laser light by the increasing depth of fluid.

**Figure 5:**
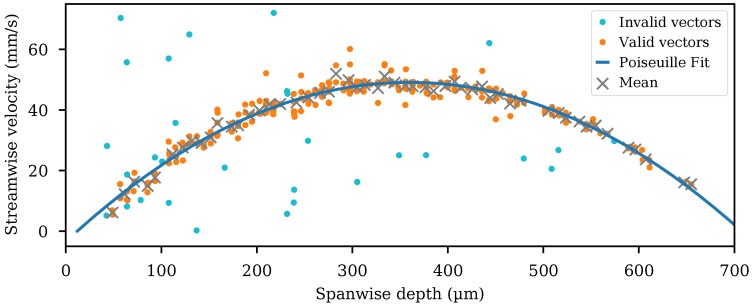
Valid and invalid flow velocity vectors shown spanwise across the microfluidic channel, for a single streamwise bin. The mean value of the valid vectors in each spanwise bin is shown. A parabolic Poiseuille fit to the valid vectors is shown. The top of the microfluidic channel as shown in Figure 2 appears at a 0 μm spanwise depth.

**Figure 6:**
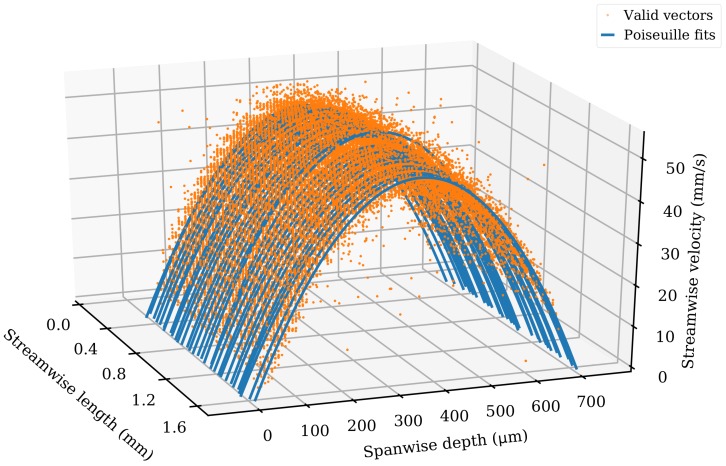
Spatially-resolved flow vectors and velocities for the straight channel microfluidic chip. Flow velocity is shown on the z-axis. The x-axis is divided into spatial bins of 10 μm. The y-axis is continuous. Orange dots represent individual valid velocity vectors, whilst blue lines represent the parabolic Poiseuille equation fits. There are 140,000 valid vectors. Only every second streamwise bin is plotted for clarity.

The velocity vectors were further divided into spanwise sections of 10 μm. The median value for each bin was determined, and median filtering [28] was applied to remove velocity vectors differing by more than 30% from the median value. It was to be expected that there was a deviation from the median in the raw time-resolved vectors due to the time-dependent effect of syringe pump pulsation (observed at ≈100 hertz by [31]), although the combined acquisition was sufficiently long that the median values were not affected. A plot of the raw and filtered velocity vectors is shown in Figure 5. The raw vectors rejected by the filtering process were labelled “invalid”, and the raw vectors that passed the filtering process were labelled “valid”. The mean value of the valid vectors was plotted for each bin. A fit to the expected parabolic Poiseuille flow equation to the valid vectors is shown. The valid vectors and Poiseuille flow fit are shown in Figure 6, a 3D plot covering the full length of the channel.

The integrated area under the Poiseuille curves multiplied by the width of the channel corresponded to the volumetric flow rate. The determined volume flow rate was 1.56 mL/min across all bins (corresponding to a peak flow velocity of 50 mm/s) with a standard deviation of 0.02 mL/min. This compared to the syringe pump setting of 1.60 mL/min. The residual error of ≈2.5% was likely due to a combination of deviation from perfect Poiseuille flow due to the (spanwise width) tapering of the channel in the dimension not imaged and the accuracy of particle position determination, which trackpy could achieve to within ≈0.5 pixels (or 1.5 μm). As shown in Figure 4, this corresponded to an expected error of ≈1 mm/s (a potential error of ≈2%) at these velocities, given 100 velocity vectors per bin [28]. However, with this instrument, the imaging was accomplished using two different beams, and therefore, it was possible that there was a systematic component of error that would not reduce as the particle number increased.

**Figure 7:**
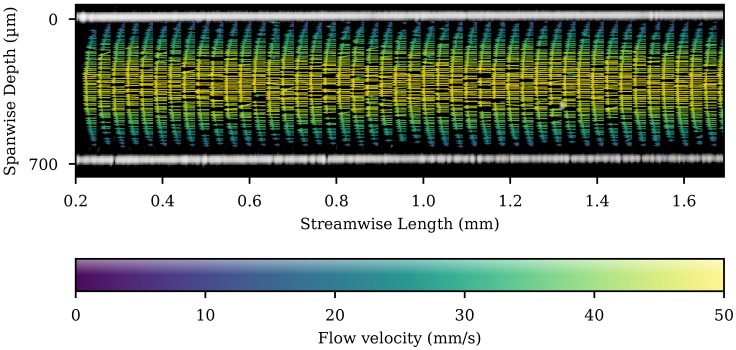
A flow velocity map is shown superimposed on a single OCT image frame from the straight section of the channel. The whole channel is shown with every third streamwise bin plotted for clarity. The bins are 10 μm square. The length of the velocity vector is additionally indicated by the colour bar. Missing arrows represent empty bins.

A flow velocity map of these data is shown superimposed on a single OCT image frame in Figure 7. The flow from a single 3.65 s measurement can be seen in the Supplementary Materials Video S1: Straight microfluidic chip section flow.

### 3.2. Flow Velocimetry for the Sloped Channel Section of the Microfluidic Chip

**Figure 8:**
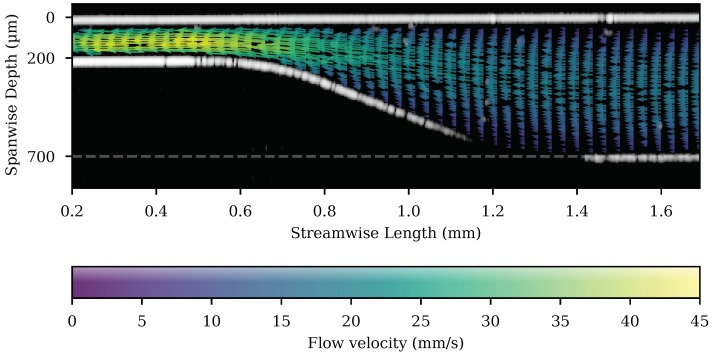
A flow velocity map is shown superimposed on a single OCT image frame from the sloped section of the channel. The whole channel is shown with every third streamwise bin plotted for clarity. The bins are 10 μm square. The length of the velocity vector is additionally indicated by the colour bar. Missing arrows represent empty bins. The grey line at 700 μm depth has been added to mark the position of the bottom of the channel on the x-axis.

Spatially-resolved flow velocimetry was also performed on a sloped microfluidic chip section. Nine sets of 3.65 s measurements were merged to a total time of 32.85 s captured over approximately 5 min duration. A flow velocity map of these data is shown superimposed on a single OCT image frame in Figure 8. The velocity of the particles increased as they moved from the wide channel (right, ≈18 mm/s, ±1; see Figure 4) to the narrow section (left, ≈41 mm/s, ±1), a ratio in the range of 2.1 to 2.5.

The area ratio of the sections of the microfluidic channel before and after the slope could be calculated based on the diagram shown in Figure 3 and the OCT measurements of the channel. The extreme limits of this ratio were given by the largest possible cross-sectional area of the deep channel (sum of A plus B, as labelled in Figure 3) over the smallest possible area of the shallow channel (labelled A in Figure 3), with the additional constraint that the bottom of the shallow channel and the top of the lower trapezium (labelled B in Figure 3) had the same dimension. This gave areas of A= 0.24 mm^2^, B= 0.50 mm^2^ for the upper ratio (A+B)/A limit of 3.1 and A= 0.27 mm^2^, B= 0.44 mm^2^ for the lower ratio (A+B)/A limit of 2.6.

The maximum measured ratio (2.5) was exceeded by the minimum area ratio (2.6), and it was speculated that two effects may contribute to explaining this. For the area, the spanwise-width constriction was not well modelled by the bottom trapezoidal shape, but instead had greater constriction at the base of the channel to support the mass resting above it [23]. For the peak velocities, the depth of the channel may cause the flow to vary differently in the spanwise-width direction, since in the shallow channel, the nearness of the top and bottom surface may dominate the forces, restraining the flow [32]. Together, these limitations emphasised the need for truly 3D flow velocimetry techniques capable of simultaneous channel measurement.

This result illustrated the flow mapping functionality of the instrument. In this case, trackpy was able to identify flow velocity vectors along the channel using a channel flow predictor starting from the expectation that particle position would primarily change in the streamwise direction. The flow from a single 3.65 s measurement can be seen in the Supplementary Materials Video S2: Sloped microfluidic chip section flow.

## 4. Discussion and Conclusions

In a prior publication, a dual beam optical coherence tomography (OCT) instrument was shown to be capable of measuring flow velocities in excess of 1 m/s [18]. In this publication, the instrument was shown to be capable of 2D spatially-resolved flow velocity measurement by reference to Poiseuille flow in a straight channel, over a spanwise depth range of 700 μm and a streamwise length of 1600 μm, with a 10 μm spatial resolution of the calculated velocity vectors, at velocities up to 50 mm/s. We also demonstrated the system with measurements of the more complex flow field in a sloped channel geometry.

This instrument retained the advantages of OCT compared to microscope-derived imaging techniques:

- requiring only a single optical access port (in this case, above the microfluidic chip),
- simultaneously imaging the structure of the microfluidic channel,
- without requiring fluorescent particles,
- providing a depth-section velocity profile (as opposed to the horizontal-section),
- at millimetre depth through a microfluidic chip.

Although a commercially available microfluidic chip was used for this work, difficulties were encountered around the deviation from specification (there was additional narrowing from a specified 0.90 mm to 0.77 mm around the base of the slope, possibly due to PMMA flow during the injection moulding process) [23], which impacted the expected flow velocity. This emphasised the need for flow measurement even in cases where commercial microfluidic chips were utilised with known pump rates and for truly 3D flow measuring instruments to capture fully and directly the flow in such situations. Approaches to provide this capability with minimal impact on the measurable flow velocities will be explored in future work.

The high 1 m/s velocity 1D measurement achieved in [18] could not be matched in 2D. Particles had to remain within the scan plane in order to be imaged by both Beam A and Beam B, so that a velocity vector could be determined for that particle. The maximum allowable spanwise width displacement (out of the scan plane) was the beam width of ≈10 μm, and the time interval between the Beam A and Beam B images was ≈1.1 ms for a slow-moving particle; hence, the maximum allowable spanwise width velocity was ≈9 mm/s. Additionally, with a well-known 1D flow profile, aggressive velocity vector filtering could be applied to recover the actual velocity [18]. In the case of a less well-defined 2D profile, it was not possible to filter aggressively. Fifty millimetres per second represented an indication (rather than a hard limit) of the velocity order-of-magnitude that could currently be measured in 2D. A full 3D system requiring significant development of the scanning method and processing algorithm would be needed to overcome this limitation.

The spatial resolution of the calculated velocity vectors and the resolution of the magnitude of those velocity vectors depended primarily on the separation of the beams and the velocity of the beam scan. For slow moving particles, tracking could be performed using consecutive frames from Beam A (although no such analysis was included herein). This would reduce the tolerable spanwise width velocity and would potentially add considerable complexity in managing the interface between the single-beam-tracking and dual-beam-tracking regimes. In the ideal case, a series of beams would provide more complete information, but this raises considerable fibre optic and data processing difficulties. It is intended to explore adding more OCT beams in future work.

The trackpy software was the limitation in applying particle tracking to a more varied set of flow situations. In part, this was due to the mismatched delay between A-to-B images compared to B-to-A images (a feature shared with double exposure camera-based PIV [28]) and in part because of misalignment between the dual beams, meaning particles did not necessarily appear in subsequent images. In future work, it is intended to develop bespoke software optimised to the instrument that determines flow velocity from the OCT images. Either with custom tracking software optimised for OCT images or alternatively by the use of correlation based particle imaging velocimetry techniques, the need to track individual particles will be eliminated.

## Supplementary Materials

The following are available online at 10.17862/cranfield.rd.1152251 from the Cranfield Online Research Data Repository or https://www.mdpi.com/2072-666X/11/4/351/s1: Video S1: Straight microfluidic chip section flow. Video S2: Sloped microfluidic chip section flow. Data and analysis code used to generate Figure 5, Figure 6, Figure 7 and Figure 8.

## Abbreviations

The following abbreviations are used in this manuscript:

OCT	Optical coherence tomography
PIV	Particle image velocimetry
PMMA	Polymethyl methacrylate
